# A study on the correlation between knee muscle strength and agility in competitive Wushu Changquan athletes

**DOI:** 10.3389/fphys.2026.1736668

**Published:** 2026-02-11

**Authors:** Liu Zhiyong, Li Weitang, Syed Ghufran Hadier, Zhou Xiaoyuan, Yi Hokun

**Affiliations:** 1 Department of Physical Education Teaching and Research, Xinjiang University, Urumqi, China; 2 School of Physical Education, Kunsan National University, Gunsan, Republic of Korea; 3 Department of Sports Sciences, Bahauddin Zakariya University, Multan, Pakistan; 4 School of Physical Education, Shanxi University, Taiyuan, China; 5 University of Science and Technology Beijing, Beijing, China

**Keywords:** agility, Changquan (Long Fist), competitive Wushu, isokinetic strength, knee joint strength

## Abstract

**Background:**

Changquan (Long Fist) is a competitive martial arts discipline characterized by complex offensive and defensive techniques requiring high levels of coordination, agility, and strength. The knee joint, as the central link between the upper and lower limbs, plays a vital role in generating power and maintaining movement stability. This study investigated the relationship between knee joint muscle strength and agility among competitive Changquan athletes.

**Method:**

A mixed-method design involving literature review, expert interviews, questionnaire survey, and experimental testing was employed. Twelve male Changquan athletes underwent bilateral isokinetic knee strength assessments at angular velocities of 60°/s (maximal strength) and 240°/s (explosive strength) using a Biodex System 4 Pro dynamometer. Peak torque (PT), relative peak torque (PT/BW, %BW), flexor–extensor ratio (F:E), total work (TW), and endurance ratio (ER) were recorded. Agility was evaluated using the T-test and 15 s push-up test. Pearson correlation analysis examined relationships between strength and agility parameters.

**Results:**

Results showed that knee extensor strength exceeded flexor strength, with mean peak torque values of 236.39 ± 17.62 N·m and 131.99 ± 13.54 N·m at 60°/s, and 133.85 ± 12.47 N·m and 97.85 ± 10.61 N·m at 240°/s. The right side was slightly stronger, though differences were not significant (p > 0.05). Athletes achieved excellent agility scores (T-test: 9.31 ± 0.16 s; push-ups: 9.66 ± 0.89 reps). Strong positive correlations were found between agility and slow flexor peak torque (r = 0.699, p = 0.011), slow extensor relative peak torque (r = 0.578, p = 0.049), and total work of slow flexors (r = 0.619, p = 0.032). The degree of correlation with agility followed the order: maximal strength > explosive power > strength endurance.

**Conclusion:**

Competitive Wushu Changquan athletes possess relatively balanced bilateral knee muscle strength; however, the flexor muscles are comparatively weaker, which may increase the risk of sports injury. Knee joint muscle strength particularly maximal and explosive flexor capacity is a key determinant of agility in competitive Wushu Changquan athletes. Balanced enhancement of flexor and extensor strength is recommended to improve performance efficiency, technical execution, and injury prevention. These findings provide a scientific basis for optimizing strength and conditioning programs for Changquan athletes through targeted flexor–extensor development to improve agility, performance precision, and injury prevention.

## 1 Introduction

Wushu Changquan (Long Fist) is a competitive martial art characterized by extensive, high-velocity movements sequences that demand exceptional technical precision, expressive control, and aesthetic presentation (Mroz, 2011). As a performance discipline, it synthesizes athletic technique, skill execution, and artistic interpretation, embodying the integration of strength, agility, and rhythmic coordination that defines competitive martial arts (Dong, 2024). Athletes execute self-choreographed routines combining standardized techniques with individual creativity, including jumping-based maneuvers that demonstrate power, balance, control, and coordination elegance (Cheng and Guo, 2024). These acrobatic and rotational actions rely on coordinated knee flexor–extensor mechanics to generate lift and safely absorb impact on landing. Consequently, optimal lower-limb strength is essential for performance efficiency and injury prevention (Zhu and Liu, 2024). Frequent postural transitions and multidirectional position changes further require the knee to remain in a semi-flexed posture, imposing sustained demands on stability, endurance, and neuromuscular control throughout performance.

Wushu Changquan performance depends on the ability to generate and regulate force rapidly during rapid postural transitions and multidirectional movement involving high-velocity jumps, explosive take-offs and landings that place substantial stress on the knee joint (Chen and Morazuki, 2025). In this context, efficient change of direction (COD) capability integrates muscular strength, balance, and neuromuscular coordination, allowing athletes to decelerate, stabilize, and re-accelerate with precision, has become a key focus in performance analysis (Falch et al., 2022). Consequently, agility and lower-limb strength are fundamental determinants of Changquan performance, as they underpin the athlete’s ability to execute quick directional shifts and posture adjustments with both efficiency and artistry.

Studies in related sports such as taekwondo, gymnastics, and fencing have shown significant evidence that knee muscle strength correlates with agility and COD performance (Zemková et al., 2013; Kovacikova and Zemková, 2021). However, the specific muscle groups and isokinetic parameters most predictive of agility remain inconsistent across studies, partly due to differences in angular velocities, test protocols, and athlete populations (Li et al., 1996). Despite biomechanical similarities within these sports, evidence describing the strength and agility relationship in Wushu Changquan remains limited. Understanding this relationship is particularly important because Wushu Changquan involves aerial spins, cyclic jumps, and rapid balance transitions that challenge neuromuscular coordination, dynamic stability, and motor control, while also imposing repetitive mechanical loads that may increase the risk of overuse injuries when flexor–extensor imbalances exist (Ghafouri et al., 2020; Wang et al., 2024). Quantifying isokinetic muscle function enables identification of such asymmetries and informs targeted training to enhance stability, agility, and injury resilience.

Although isokinetic dynamometry is widely used to assess lower-limb strength in sport science, few studies have profiled Changquan athletes or examined how their knee strength characteristics relate to agility outcomes. Most existing work emphasizes technical or aesthetic evaluation rather than physiological determinants of skill execution. Addressing this gap can support evidence-based conditioning and preventive strategies specific to martial-arts performance.

Accordingly, this study aimed to (1) characterize isokinetic knee strength characteristics of elite Changquan athletes at two angular velocities; 60°/s (maximal strength) and 240°/s (power/endurance), and (2) examine their associations with agility performance assessed through standardized COD testing. A secondary objective was to evaluate bilateral differences and flexor–extensor ratios to determine whether muscular balance contributes to superior agility performance. The findings will enhance understanding of lower-limb biomechanics in martial arts specifically Changquan and guide scientific approaches to performance optimization and injury prevention among elite competitive Wushu athletes.

### 1.1 Significance of knee muscle strength and agility

Knee joint muscle strength forms the biomechanical foundation for executing technical movements, whereas agility influences bodily coordination and movement control during rapid transitions. Identify the correlation between these two factors and identify key muscle strength elements that significantly impact agility enabling targeted training to improve both movement efficiency and technical precision. Within the Changquan (Long Fist) discipline, this relationship not only enhances understanding of sport-specific biomechanics but also contributes to the theoretical framework of performance optimization and evidence-based conditioning.

### 1.2 Theoretical and applied value

Systematic analysis of the relationship between knee joint muscle strength and agility extends interdisciplinary knowledge in fields such as martial arts science, biomechanics, and sports training. It supports the development of specialized fitness evaluation and monitoring systems tailored to martial arts performance. This research deepens the scientific understanding of neuromuscular coordination, mechanical load distribution, and energy efficiency in acrobatic movements. Practically, it guides coaches in identifying critical strength indicators for improvement, facilitating the refinement of individualized training protocols that enhance agility performance, movement execution quality, and competitive performance.

## 2 Literature review

### 2.1 Research on competitive Wushu routines

Modern competitive Wushu evolved from traditional martial arts after the founding of the People’s Republic of China and was first formalized as a national competition event in 1957. By 1959, the National Youth Wushu Games defined its developmental principles as high, difficult, beautiful, and innovative (Yi, 1999). This framework integrated technical difficulty, movement quality, and aesthetic presentation within a standardized scoring system. Over time, a structured competitive system encompassing events such as Changquan (Long Fist), Daoshu (Sword), Jianshu (Saber), Gunshu (Staff), Qiangshu (Spear), Nanquan (Southern Fist), and Taijiquan (Tai Chi) gradually emerged (Gao and Soonjan, 2024). With continuous refinement of event standards and competition rules, competitive Wushu has entered the international sporting arena, including its inclusion in the Youth Olympic Games (Kinoe and Ikeuchi, 2023).

Among its disciplines, Changquan (Long Fist) remains a central event, characterized by extended, rhythmic, and acrobatic movements that combine upper-body flexibility with lower-body stability, strength, and agility. These technical elements demand high lower-limb strength, postural control, and rapid coordination to sustain stability and expressiveness during dynamic routines (Wang et al., 2024). Hence, lower-limb strength, particularly at the knee joint, is critical for stability and expressive control during both take-off and landing phases.

Muscle strength represents the capacity of muscles, under neural regulation, to generate and transmit force efficiently. Knee strength, primarily derived from the quadriceps and hamstrings, supports stability, movement control, and impact absorption during high-intensity techniques (Liu and Qu, 2022). In Changquan, particularly during high-difficulty aerial actions such as the 720° Whirlwind Kick, exceptional lower-limb strength enables optimal jump height and controlled landing. Balanced flexor–extensor development ensures joint integrity and efficient energy transfer (Zhuang, 2001).

### 2.2 Studies on knee joint muscle strength

In competitive martial arts routines, strength is the core element for executing various static and dynamic movements. This is particularly true for high-difficulty jumping maneuvers like the 720° Whirlwind Kick or 720° Spinning Turn, which demand exceptional lower-body explosiveness. Strong muscle strength not only enhances jump height and movement stability but also determines the quality of transitions and execution (García-García et al., 2013). Only athletes with exceptional muscle strength can achieve seamless movement connections and overall technical excellence. Beyond isolated strength assessment, recent research increasingly supports the use of standardized reference frameworks and cross-validated normative models to interpret isokinetic outcomes and contextualize athlete performance across developmental stages and competitive levels (Hadier et al., 2024a; Hamdani et al., 2023; Long et al., 2024).

Knee joint muscle strength is commonly assessed using isokinetic testing methods (Gaines and Talbot, 1999). Compared to isometric and isotonic testing, isokinetic strength testing controls movement through constant angular velocity, enabling accurate measurement of concentric and eccentric muscle force throughout the full range of motion while ensuring safety. Devices such as the Biodex System 4 and ISOMED 2000 allow assessment of concentric and eccentric muscle actions under constant angular velocity, yielding indicators including peak torque (PT), relative peak torque (PT/BW), flexor-to-extensor ratio (F:E), total work (TW), and endurance ratio (ER) (Wilk et al., 2024; Dvir, 2025).

The selection of angular velocity directly impacts testing objectives and outcomes: Lower angular velocities 30°–60°/s are typically used for slow-speed testing, suitable for assessing maximum muscle strength; whereas 180°–300°/s are commonly employed for fast-speed testing, better suited for evaluating muscle power and endurance (Liu and Qu, 2022; García-García et al., 2013). Studies indicate that 60°/s is an optimal angular velocity for slow-speed testing, effectively reflecting maximum muscle output, while fast-speed testing at 180°/s or higher evaluates fast-twitch muscle fiber contraction capacity and muscle fatigue levels (Guoping et al., 1988). The number of repetitions also varies typically 4–6 for low-speed tests and 20–30 for high-speed protocols to balance measurement precision with fatigue prevention (Li, 2022). Appropriate selection of speed and repetition count enhances data reliability and minimizes injury risk. Collectively, these indicators provide a comprehensive representation of muscular performance, forming a scientific foundation for individualized training and rehabilitation planning.

### 2.3 Studies on knee muscle strength in other sports

Research across various sports consistently highlights the significance of knee muscle strength for lower-limb stability and performance (Lv et al., 2026). Research indicates that fencers exhibit insufficient knee extensor explosive power and reduced fatigue resistance, highlighting the need for endurance-specific conditioning (Peek et al., 2022; El-Ashker et al., 2022). Among winter sports approximately 35% of athletes exhibit both knee strength imbalances, with gender-based strength differences lacking statistical significance (El-Ashker et al., 2022). In water polo, peak torque serves as a key indicator of muscular work capacity (Ren et al., 2025). Basketball research reveals that female players produce lower PT values at high angular velocities, with position-based strength differences necessitating targeted training (Wang et al., 2025). Rock climbers demonstrate optimal flexor strength angles between 136° and 156°, with training within this range yielding higher results (Bobowik et al., 2023). Aerobics athletes exhibit issues such as low H/Q ratios, necessitating enhanced targeted strength training (Kyselovičová et al., 2023). For wrestlers, flexor strength increases significantly with rising angular velocity (Baić et al., 2022). These findings collectively confirm that balanced knee strength underpins lower-limb stability, explosive capacity, and agility across diverse sports contexts.

### 2.4 Agility: concept and assessment

Agility refers to an athlete’s capacity to rapidly and efficiently alter body position or direction in response to environmental stimuli. It involves integrated neural and muscular control that enables quick acceleration, deceleration, and redirection of movement (Yao and Niu, 2024). In sports science, agility is often conceptualized through three dimensions; movement transition, directional change, and reactive response each governed by neuromuscular coordination and motor planning (Pietraszewski et al., 2021).

Agility testing incorporates both physical and cognitive components to capture multidimensional performance. Standard field assessments include: the T-test, Illinois Agility Test, and Reactive Light or Visual Stimulus tests. Zhao Xitang’s three-factor model of agility categorizes agility into three factors; directional change, movement transition, and coordination, provides a theoretical foundation for quantifying these dimensions. Such assessments are widely applied in performance sports, including martial arts, where athletes must integrate rapid movement control with precision and aesthetic expression (Si et al., 2025; Zhiping et al., 2011).

## 3 Methods

### 3.1 Study design

**FIGURE 1:**
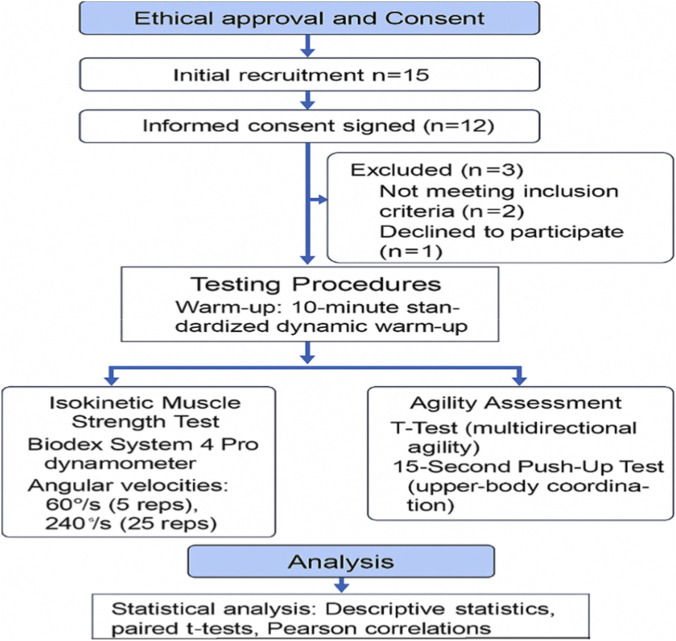
Flow diagram.

This cross-sectional study examines the relationship between knee joint isokinetic strength indicators and agility performance among elite Wushu Changquan athletes. The research was conducted at the Sports Biomechanics Laboratory of Wushu Training Hall, Beijing Sport University, between 15–20 October 2022. The design was chosen to enable assessment of multiple physiological variables such as peak torque, torque ratios, total work, and agility indicators under controlled laboratory and field-based testing conditions. Figure 1 shows the study process.

### 3.2 Participants

To investigate the relationship between knee joint muscle strength and agility performance among male elite-level Wushu Changquan (Long Fist) athletes, a purposive sampling strategy was employed. A total of 15 eligible elite athletes were recruited from the National Wushu School at Beijing Sport University within a centralized training environment. Of these, 12 athletes voluntarily agreed to participate and completed all testing procedures.

The final sample consisted of athletes aged 21.75 ± 2.34 years, with a height of 172.33 ± 4.29 cm, body mass of 65.17 ± 5.31 kg, and training experience of 13.75 ± 2.80 years (see Appendix Section A3). The sample size was constrained by the limited availability of elite Changquan athletes and the demanding nature of isokinetic testing. Similar sample sizes have been reported in previous biomechanical and isokinetic studies involving elite martial arts and combat sport athletes, where participant homogeneity is prioritized over larger sample numbers (El-Ashker et al., 2022; Wilkosz et al., 2021).

### 3.3 Inclusion and exclusion criteria

All participants were required to meet specific inclusion and exclusion criteria to ensure sample homogeneity and safety during testing. Eligible participants were required to have Changquan as their primary discipline and to be national championship level athletes with a minimum of 10 consecutive years of martial arts training experience. Only athletes who were injury-free for at least 6 months prior to data collection and medically cleared by team medical staff for high-intensity physical assessments were included in the study. Before testing, all eligible athletes were briefed on the study objectives and procedures to ensure understanding of the testing process and equipment. Testing conditions were strictly standardized, with participants maintaining their usual daily routines to minimize potential data variability. All participants provided written informed consent prior to participation.

### 3.4 Selection of indicators

To establish a comprehensive and evidence-based framework for selection of methods and indicators, the study adopted a three-stage methodological sequence integrating literature analysis, expert consultation, and questionnaire investigation. First, a literature review identified theoretical indicators and constructs related to knee joint muscle strength, agility, and technical performance in martial arts and comparable sports. Second, an expert interview method was employed to validate and refine the selected indicators for their scientific adequacy and practical applicability within the Changquan performance context. Multiple experts in martial arts and physical fitness were invited for interviews for test indicators selection (see Supplementary Appendix Section A1,2 for details). Basic information about the experts interviewed is provided in Table 2. Discussions covered knee joint muscle strength testing metrics, agility assessment methods, and other topics to enhance the scientific rigor and practicality of the research design. Finally, a questionnaire survey was distributed to coaches, athletes, and sport science professionals to quantify the perceived importance, reliability, and applicability of the selected indicators, thus providing empirical support for the proposed analytical framework. This multi-method design ensured theoretical grounding, expert confirmation, and quantitative verification of the study variables (Table 1).

**TABLE 1:** Common parameters for constant-speed isokinetic testing.

Indicator	Definition/Application
Peak torque (PT)	Maximum rotational force output during muscle contraction; considered the gold standard for isokinetic strength testing
Relative peak torque (PT/BW)	PT normalized to body weight; enables comparison across athletes
Flexor-extensor ratio (F:E)	Ratio between flexor and extensor torque, reflecting joint muscle strength balance
Angle of peak torque (AOPT)	Joint angle at which PT occurs reflects mechanical advantage
Total work (TW)	The maximum workload during repeated muscle contractions, used to evaluate muscle endurance capacity
Endurance ratio (ER)	The ability of muscle to tolerate fatigue during repeated contractions

**TABLE 2:** Basic information of experts interviewed.

Name	Gender	Title	Employer
He	Female	Professor	Beijing Sport university
Chen	Female	Associate professor	Beijing Sport university
Wang	Male	Professor	Beijing Sport university
Wei	Male	Associate professor	Beijing Sport university

Participants were excluded if they presented any acute or chronic musculoskeletal injury, were undergoing rehabilitation, or were taking medication that could affect neuromuscular function or physical performance. Additionally, athletes who failed to complete all testing procedures according to the standardized protocol were excluded from the final analysis.

### 3.5 Data collection procedures

All data collection procedures were conducted under standardized laboratory under controlled laboratory conditions at a room temperature of approximately 22 °C to ensure accuracy and reliability. Prior to testing, each athlete completed a 10 min standardized warm-up that included light jogging, dynamic stretching, and two submaximal practice trials to ensure familiarization trials before the main tests began.

Testing began with isokinetic knee strength assessment, conducted first on the dominant limb and then on the non-dominant limb. Both flexion and extension were measured at two angular velocities: 60°/s to assess maximal muscle strength and 240°/s to evaluate muscular power and endurance. A 1 min rest was provided between limbs and a 3 min interval between the two speed protocols. After a recovery period of 10 min, participants completed the agility tests, beginning with the T-test and followed by the 15 s push-up test. All athletes were tested individually and supervised by trained evaluators to ensure procedural consistency and accuracy. Each test was performed twice, with the best valid result used for analysis.

### 3.6 Measures

#### 3.6.1 Knee joint muscle strength testing

**FIGURE 2:**
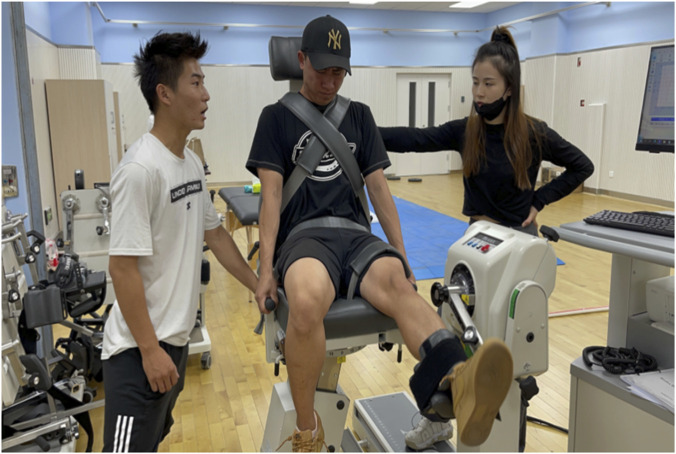
Knee joint muscle strength test.

Knee joint muscle strength was measured using the Biodex System 4 Pro Isokinetic Dynamometer (Biodex Medical Systems Inc., United States). Participants were seated with their hips at 90° of flexion and secured with straps at the trunk, pelvis, and thigh. The lateral femoral epicondyle was aligned with the dynamometer’s axis of rotation, and the lever arm pad was positioned approximately 2 cm above the lateral malleolus (See Figure 2).

The testing protocol comprised two angular speed conditions. The first, low-velocity testing (60°/s, 5 repetitions), measured maximal voluntary torque to determine peak torque (PT) and relative peak torque (PT/BW, %BW). The second, high-velocity testing (240°/s, 25 repetitions), evaluated total work (TW), endurance ratio (ER), and the flexor–extensor ratio (F:E).

The parameters were defined as follows: PT (Nm) represented the maximum torque generated during a single repetition; relative peak torque (PT/BW, %BW) was calculated as peak torque normalized to body weight and expressed as a percentage of body weight; the flexor–extensor ratio (F:E, %) represented the hamstring-to-quadriceps torque ratio; total work (TW, J) represented the cumulative mechanical work performed; average power (AP, W) described the mean work performed per unit time; and the endurance ratio (ER, %) quantified fatigue resistance, calculated as (final TW ÷ initial TW) × 100.

The selection of these two testing velocities followed standard isokinetic protocols, with 60°/s representing maximal strength output and 240°/s reflecting muscular power and endurance capacities. Adequate rest intervals, 1 min between limbs and 3 min between testing velocities were provided to minimize fatigue and ensure data reliability.

### 3.7 Agility assessment

**FIGURE 3:**
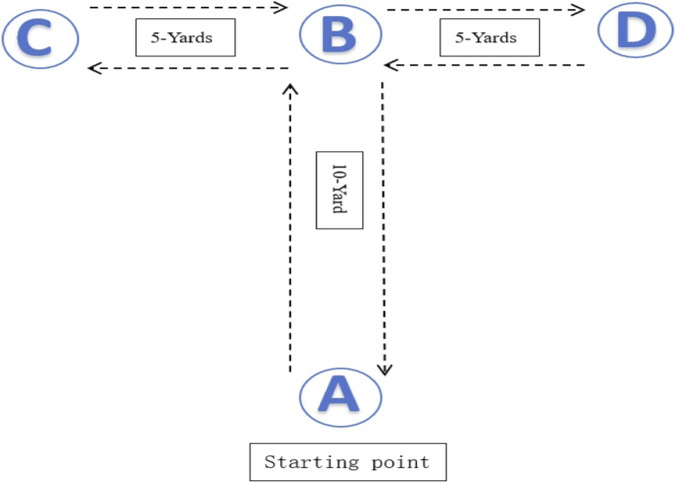
T-test.

Agility performance was measured using two standardized field-based assessments. The T-test evaluated multidirectional change-of-direction ability (For testing procedure see Figure 3). Participants sprinted forward 9.14 m, shuffled 4.57 m laterally to both sides, and then backpedaled to the start line. Performance time was recorded with electronic timing gates (Smart-Speed, Fusion Sport, Australia), and lower time values indicated better agility.

**FIGURE 4:**
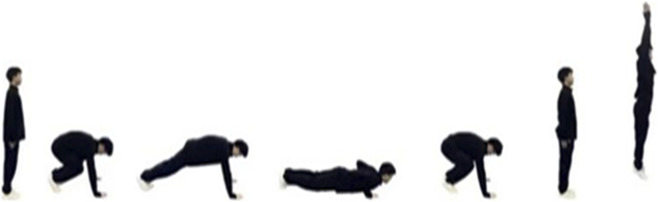
Push-ups.

The 15 s push-up test assessed upper-body coordination and control during rapid movements (For testing procedure see Figure 4). Participants completed as many correctly performed push-ups as possible within 15 s, with each repetition supervised and verified by trained coaches. Both tests were performed after a standardized 10 min warm-up and two familiarization trials. The best valid performance from each test was used for analysis.

### 3.8 Statistical analysis

All statistical analyses were performed using SPSS version 26.0 (IBM Corp., Armonk, United States of America). Descriptive statistics were calculated for all variables and expressed as mean ± standard deviation (SD). Data normality was verified using the Shapiro–Wilk test, while homogeneity of variances was assessed using Levene’s test. Differences in isokinetic variables between the dominant and non-dominant limbs were examined using independent-samples t-tests. To evaluate the relationships between isokinetic strength indicators (PT, PT/BW, F:E, TW, AP, and ER) and agility performance outcomes (T-test time and push-up repetitions) were evaluated using correlation analyses. Pearson product–moment correlation coefficients were applied for normally distributed variables, whereas Spearman’s rank-order correlations were used when normality assumptions were violated.

All included participants completed the full testing protocol, and no missing data was present in the final dataset. Therefore, a complete-case analysis approach was applied, and no data imputation procedures were required. Statistical significance was set at p < 0.05 (two-tailed). To aid interpretation, T-test completion times were reverse-coded so that higher values reflected superior agility performance. Effect sizes (Cohen’s d) were reported for limb comparisons, with values of 0.2, 0.5, and 0.8 indicating small, medium, and large effects, respectively. Correlation coefficients were interpreted as weak (r < 0.30), moderate (0.30–0.50), or strong (r > 0.50). A *post hoc* power analysis indicated that for a correlation coefficient of r ≥ 0.65, with α = 0.05 and a sample size of n = 12, the achieved statistical power was 0.81 (G*Power version 3.1), supporting adequate sensitivity for detecting large associations.

### 3.9 Ethics approval

This research was approved by the Institutional Review Board of Beijing Sport University (Approval No. BSU-2022-09-K). The study adhered to the Declaration of Helsinki for human research. All participants were adults, informed of the study objectives, procedures, and potential risks, and provided written consent prior to testing. Data confidentiality was strictly maintained, and participation was voluntary with the right to withdraw at any stage.

## 4 Results

### 4.1 Knee muscle strength in competitive Wushu Changquan athletes

Table 3 presents the results of isokinetic strength testing for knee flexors and extensors at two angular velocities (60°/s and 240°/s). Results show that in isokinetic strength testing, peak torque values decreased with increasing angular velocity. Test results indicate that at 60°/s the peak torque of the right knee extensors and flexors was 236.39 N·m and 131.99 N·m, respectively, while the left side recorded 231.43 N·m and 129.01 N·m. At 240°/s, the right-side extensors and flexors reached 133.85 N·m and 97.85 N·m, and the left side 130.71 N·m and 96.71 N·m, respectively. At both velocities, extensor strength was greater than flexor strength, and no significant bilateral difference was found (p > 0.05).

**TABLE 3:** Isokinetic knee strength parameters at two angular velocities (60°/s and 240°/s).

Angular velocity (°/s)		Specification (n = 12)	Peak torque (N·m) Mean ± SD	Relative peak torque (%BW) Mean ± SD	Peak torque angle (°) Mean ± SD
60°/s	Extensor	Right side	236.39 ± 32.37	364.69 ± 48.03	68.75 ± 6.52
		Left side	231.43 ± 30.62	356.41 ± 40.55	67.83 ± 11.04
		*t*-value (*p*-value)	0.385 (0.704)	0.456 (0.653)	0.248 (0.807)
	Flexor	Right side	131.99 ± 14.28	203.99 ± 23.47	27.75 ± 8.23
		Left side	129.01 ± 16.92	199.04 ± 25.75	26.25 ± 11.09
		*t*-value (*p*-value)	0.467 (0.645)	0.492 (0.627)	0.376 (0.710)
240°/s	Extensor	Right side	133.85 ± 17.08	209.51 ± 34.79	71.58 ± 7.05
		Left side	130.71 ± 14.36	201.15 ± 18.07	69.00 ± 10.22
		*t*-value (*p*-value)	0.488 (0.630)	0.739 (0.468)	0.721 (0.479)
	Flexor	Right side	97.85 ± 9.50	150.03 ± 13.03	30.09 ± 9.59
		Left side	96.71 ± 9.74	149.58 ± 13.06	29.33 ± 10.87
		*t*-value (*p*-value)	0.291 (0.774)	0.086 (0.932)	0.179 (0.859)

Data are presented as Mean ± SD (n = 12); PT, peak torque; PT/BW, Peak Torque expressed as a percentage of body weight (%BW); N·m, Newton-metre (unit of torque); %BW, percentage of body weight; Angle of PT, knee joint angle at which maximum torque occurred; *p*, Probability value (significance level); °, Degrees.

The relative peak torque (PT/BW) also showed a decreasing pattern with increasing angular velocity. At 60°/s, right-side extensor and flexor values were 364.69% BW and 203.99% BW, while left-side values were 356.41% BW and 199.04% BW. At 240°/s, right-side values were 209.51% BW and 150.03% BW, while left-side values were 201.15% BW and 149.58% BW, showing a slight advantage for the right side but still without significant difference (P > 0.05).

The peak torque angle increased slightly with higher angular velocity. For extensors, the right limb peaked between 68° and 72° and the left between 67° and 69°; for flexors, the right and left peaks occurred between 27°–30° and 26°–29°, respectively. Bilateral angular differences were not statistically significant (p > 0.05), confirming consistent joint mechanics between limbs.

Table 4 results show that the flexor–extensor (F:E) peak torque ratio increased with angular velocity in both limbs. At 60°/s, the right and left limbs recorded mean ratios of 56.06% ± 6.16% and 55.80% ± 5.97%, respectively. At 240°/s, the ratios increased to 74.04% ± 5.05% and 73.29% ± 4.88%, respectively. No significant difference was found between limbs across different angular velocities (p > 0.05). These findings demonstrate symmetrical coordination between flexor and extensor muscle groups, indicating balanced muscular development and stable knee-joint control during movement execution.

**TABLE 4:** Flexor–extensor ratio Peak Torque Ratios at two angular velocities (60°/s and 240°/s).

Angular velocity (°/s)	F:E ratio (%) Mean ± SD		*t*-value	*p*-value
60°/s	Right side	56.06 ± 6.16	0.275	0.786
	Left side	55.38 ± 6.02		
240°/s	Right side	74.04 ± 5.05	−0.062	0.951
	Left side	73.87 ± 8.44		

Data are presented as Mean ± SD (n = 12); F:E, flexor–extensor; *p*, Probability value (significance level); °, Degrees.

The knee joint efficiently converts chemical energy into mechanical energy during rapid movements, with total work reflecting muscle performance capacity per unit time. Table 5 results show that, for competitive Wushu Changquan athletes, total work performed by the knee extensors exceeded that of the flexors at both testing speeds (60°/s and 240°/s). At 60°/s, mean total work for the extensors was 985.93 ± 160.32 J (right) and 967.98 ± 247.57 J (left), whereas the flexors produced 679.95 ± 135.63 J (right) and 624.89 ± 106.28 J (left). At 240°/s, total work of the extensors increased to 2918.09 ± 473.39 J (right) and 2733.31 ± 423.70 J (left), while the flexors reached 2273.83 ± 262.83 J (right) and 2241.02 ± 273.85 J (left). Across all conditions, extensors consistently generated greater work than flexors, and the right side displayed slightly higher output than the left; however, these differences were not statistically significant (p > 0.05), indicating balanced bilateral muscle strength and functional symmetry.

**TABLE 5:** Knee joint total work and endurance ratios at two angular velocities.

Angular velocity (°/s)	Indicator		Total energy (J) Mean ± SD	*t*-value	*p*-value
60°/s	Extensor muscles	Right side	985.93 ± 160.32	−0.211	0.835
		Left side	967.98 ± 247.57		
	Flexor muscles	Right side	679.95 ± 135.63	1.107	0.280
		Left side	624.89 ± 106.28		
240°/s	Extensor muscles	Right side	2918.09 ± 473.39	1.008	0.325
		Left side	2733.31 ± 423.70		
	Flexor muscles	Right side	2273.83 ± 262.83	0.299	0.767
		Left side	2241.02 ± 273.85		
Endurance ratio (%)					
240°/s	Extensor muscles	Right side	0.58 ± 0.11	0.889	0.383
		Left side	0.54 ± 0.06		
	Flexor muscles	Right side	0.56 ± 0.10	0.071	0.944
		Left side	0.56 ± 0.07		

Data are presented as Mean ± SD (n = 12). t- and p-values represent paired-sample comparisons between right and left limbs. No statistically significant bilateral differences were observed (p > 0.05). Total Energy (J) reflects cumulative mechanical work performed by the knee flexor and extensor muscles at angular velocities of 60°/s and 240°/s. Endurance Ratio (ER) indicates the relative capacity of muscles to resist fatigue during repeated contractions, calculated as the ratio of final to initial total work.

As angular velocity increased from 60°/s to 240°/s, total work showed an upward trend, primarily because although maximal torque decreased, the time required to complete each repetition shortened more markedly. This resulted in greater work per unit time and reflected enhanced mechanical efficiency during high-speed contraction (21).

At 240°/s, the endurance ratio (ER) of the right knee extensors was 0.58 ± 0.11, compared with 0.54 ± 0.06 on the left. For the flexors, ER values were 0.56 ± 0.10 (right) and 0.56 ± 0.07 (left). None of these differences were statistically significant (p > 0.05). However, a directional pattern was evident: the right extensors demonstrated higher fatigue resistance than their ipsilateral flexors, while on the left side, flexors exhibited greater endurance than extensors. This suggests that right extensors and left flexors possess relatively superior fatigue resistance, reflecting mild lateralization and functional differentiation between limbs. Such side-dominant adaptations are likely influenced by habitual training asymmetries and movement mechanics specific to Changquan performance.

### 4.2 Analysis of agility traits in competitive Wushu Changquan athletes

Agility performance was evaluated using the T-test and 15 s push-up test, both of which assess multidirectional movement control and upper-body coordination. As Table 6 results show that athletes achieved a mean T-test completion time of 9.31 ± 0.16 s, indicating excellent change-of-direction speed. The mean number of push-ups completed within 15 s was 9.66 ± 0.89 repetitions, reflecting a high level of dynamic upper-body control and endurance. Across all participants, performance variation was minimal, suggesting consistent neuromuscular coordination and technical proficiency within this elite group. No statistically significant gender or limb-dominance differences were observed in agility outcomes (p > 0.05). The results indicate that competitive Wushu Changquan athletes possess well-developed agility traits characterized by rapid response ability, efficient body control, and stable execution during rapid postural transitions.

**TABLE 6:** Agility test results of competitive Wushu Changquan athletes.

Test metric	T-test (seconds)	Sit-ups (reps)
Test results	9.31 ± 0.16	9.66 ± 0.89

Values represent mean ± standard deviation for n = 12 athletes.

### 4.3 Correlation analysis between knee muscle strength and agility performance

Table 7 shows that analyzing the correlations between peak knee joint torque at different angular velocities and agility indicators for competitive Changquan martial arts athletes. The analysis showed that peak torque of the right and left knee flexors at both 60°/s and 240°/s was significantly correlated with T-test performance (r = 0.600–0.655, p < 0.05). Similarly, total work of the right and left knee flexors at 60°/s exhibited significant positive correlations with T-test results (r = 0.619 and r = 0.600, respectively; p < 0.05). These findings indicate that greater knee flexor strength and work capacity are associated with faster change-of-direction performance.

**TABLE 7:** Correlation analysis between isokinetic knee muscle strength and agility performance.

Test metrics		Peak torque at 60°/s angular velocity				Peak torque at 240°/s angular velocity			
		Right extension	Left extension	Right flexion	Left flexion	Right extension	Left extension	Right flexion	Left flexion
T-test	*r*	0.277	0.516	0.600*	0.655*	0.201	0.488	0.636*	0.618*
	*p*	0.384	0.086	0.039	0.021	0.530	0.107	0.026	0.032
Sit-up	*r*	0.400	0.288	0.365	0.159	0.494	0.098	0.231	0.217
	*p*	0.198	0.364	0.243	0.622	0.103	0.762	0.471	0.498

n = 12; * indicates significant at p < 0.05.

In addition, the *relative peak torque* of the right knee extensors at both 60°/s and 240°/s demonstrated significant positive correlations with push-up performance (r = 0.699 and r = 0.578, respectively; p < 0.05), suggesting that extensor strength contributes to explosive upper-body stabilization during rapid movement transitions.

The *flexor–extensor torque ratio* at 60°/s showed a moderate negative correlation with push-up performance (r = −0.633, p = 0.027), indicating that excessive extensor dominance may reduce coordination efficiency during complex movement execution. *Endurance ratio* values exhibited moderate but non-significant relationships with agility indicators (p > 0.05).

Overall, the results indicate that both maximal and relative lower-limb strength particularly flexor torque and total work are important determinants of agility in Changquan performance, whereas endurance variables play a lesser role in short-duration, high-intensity agility tasks.

Table 8 shows that analysis of the correlation between relative peak knee joint torque at different angular velocities and agility indicators in competitive wushu Changquan athletes reveals: At 60°/s angular velocity, the relative peak torque of the right knee extensors exhibits a highly positive correlation with the push-up test (r = 0.699, p = 0.011). At 240°/s angular velocity, the relative peak torque of the right knee extensors shows a moderate positive correlation with the push-up test (r = 0.578, p = 0.04). p = 0.011), while at 240°/s angular velocity, the relative peak moment of the right knee extensor muscles showed a moderate positive correlation with push-ups (r = 0.578, p = 0.049).

**TABLE 8:** Correlation analysis results between knee joint relative peak moment and agility quality.

Test metrics		60°/s angular velocity relative to peak torque				240°/s angular velocity relative to peak torque			
		Right extension	Left extension	Right flexion	Left flexion	Right extension	Left extension	Right flexion	Left flexion
T-test	r	0.272	0.418	0.382	0.520	0.084	0.347	0.515	0.440
	*p*	0.392	0.177	0.211	0.083	0.795	0.269	0.087	0.152
Sit-up	r	0.699*	0.414	0.393	0.198	0.578*	0.189	0.279	0.361
	*p*	0.011	0.181	0.207	0.538	0.049	0.557	0.379	0.249

n = 12; * indicates significant at p < 0.05.

Overall, these findings demonstrate that relative knee extensor torque, particularly on the right side, is a critical determinant of agility-related performance in Changquan athletes. The results further indicate that athletes with higher isokinetic strength at both slow and fast velocities tend to perform better in agility tasks, highlighting the contribution of lower-limb power to coordinated multidirectional movement and upper-body stabilization during complex Wushu routines.

Table 9 shows that by analyzing the correlation between the knee joint’s peak torque angle at different angular velocities and agility indicators in competitive wushu Changquan athletes, the following conclusions were drawn: At 60°/s angular velocity, the peak torque angle of the left knee extensor exhibited a moderate negative correlation with the T-test (r = −0.447, p = 0.145). Since p > 0.05, the correlation coefficient lacks statistical significance. It also showed a moderate positive correlation with the push-up test (r = 0.318, p = 0.313), but again, p > 0.05 indicates the correlation lacks statistical significance. The angle at which the right knee extensor reached peak torque at 240°/s angular velocity showed a moderate positive correlation with push-ups (r = 0.391, p = 0.209). Since p > 0.05, the correlation coefficient was not statistically significant.

**TABLE 9:** Correlation analysis results between knee peak torque angle and agility.

Test metrics		60°/s angular velocity reaches peak torque angle				240°/s angular velocity reaches peak torque angle			
		Right extension	Left extension	Right flexion	Left flexion	Right extension	Left extension	Right flexion	Left flexion
T-test	*r*	−0.106	−0.447	0.006	−0.009	−0.025	−0.251	0.275	−0.234
	*p*	0.743	0.145	0.986	0.978	0.939	0.431	0.387	0.465
Sit-up	*r*	−0.188	0.318	−0.112	0.092	0.086	0.391	−0.242	0.248
	*p*	0.588	0.313	0.729	0.775	0.791	0.209	0.448	0.437

n = 12; * indicates significant at p < 0.05.

Table 10 presents the correlation between knee flexor–extensor peak torque ratios and agility performance indicators at angular velocities of 60°/s and 240°/s. Results show that at 60°/s, the right-side flexor–extensor torque ratio demonstrated a significant negative correlation with push-up performance (r = −0.633, p = 0.027), indicating that greater extensor dominance (lower flexor contribution) was associated with reduced upper-body agility and coordination during rapid movement execution. For the T-test, the right and left flexor–extensor ratios showed weak positive correlations (r = 0.308 and r = 0.469, respectively; p > 0.05), suggesting that flexor–extensor balance exerts limited influence on lower-limb change-of-direction speed.

**TABLE 10:** Correlation analysis results between peak torque ratios of knee flexor and extensor muscles and agility quality.

Test metrics		60°/s angular velocity flexor-extensor peak torque ratio		240°/s angular velocity flexor-extensor peak torque ratio	
		Right side	Left side	Right side	Left side
T-test	*r*	0.308	0.469	0.325	0.163
	*p*-value	0.330	0.124	0.303	0.614
Sit-ups	r	−0.633*	−0.143	−0.383	0.143
	*p*-value	0.027	0.658	0.220	0.657

n = 12; * indicates significant at p < 0.05.

At 240°/s, all correlations between the torque ratios and agility performance (both T-test and push-ups) were weak and statistically non-significant (p > 0.05), implying that at higher contraction speeds, torque balance exerts less impact on agility compared with overall strength capacity. Collectively, these findings highlight that muscle balance between flexors and extensors contributes meaningfully to performance precision and upper-body coordination in Changquan routines, although its effect diminishes under high-speed, power-dominant conditions.

Table 11 shows that analysis of the correlation between total knee joint work at different angular velocities and agility indicators in competitive wushu Changquan athletes reveals: Total work of the right knee flexors at 60°/s angular velocity exhibits a strong positive correlation with the T-test (r = 0.619, p = 0.032). while the total work of the left knee flexors at 60°/s showed a moderate positive correlation with the T-test (r = 0.600, p = 0.039). These results suggest that lower-limb work capacity, particularly of the flexor muscles, plays a more prominent role in agility performance under slow-speed, strength-dominant conditions (60°/s) than during high-speed movements (240°/s).

**TABLE 11:** Correlation analysis results between total knee joint work and agility quality.

Test metrics		Total work at 60°/s angular velocity				Total work at 240°/s angular velocity			
		Right extension	Left extension	Right flexion	Left flexion	Right extension	Left extension	Right flexion	Left flexion
T-test	r	0.255	0.313	0.619*	0.600*	0.312	0.504	0.576	0.518
	*p*	0.424	0.322	0.032	0.039	0.323	0.094	0.050	0.085
Sit-up	r	0.015	0.243	0.009	0.124	−0.113	−0.528	−0.212	−0.367
	*p*	0.946	0.448	0.978	0.700	0.726	0.078	0.508	0.241

n = 12; * indicates significant at p < 0.05.

Table 12 shows that analyzing the correlation between knee joint endurance ratio and agility indicators at a 240°/s angular velocity for competitive wushu Changquan athletes reveals: The endurance ratio of the right knee extensors showed a moderate negative correlation with the push-up test (r = −0.369, p = 0.238). Since the p-value (0.238) exceeded the significance threshold (p < 0.05), the correlation coefficient lacked statistical significance. The endurance ratio of the left knee extensors showed a moderate positive correlation with the T-test (r = 0.438, p = 0.154). Since p = 0.154 > 0.05, the correlation coefficient lacks statistical significance. The endurance ratio of the left knee extensors showed a moderate negative correlation with push-ups (r = −0.351, p = 0.263). Since the p-value (0.263) exceeded 0.05, the correlation coefficient lacked statistical significance.

**TABLE 12:** Correlation analysis results between knee joint endurance ratios and agility qualities.

Test metrics		240°/s angular velocity endurance ratio			
		Right extension	Left extension	Right flexion	Left flexion
T-test	r	0.028	0.438	−0.098	0.004
	*p*	0.931	0.154	0.763	0.991
Sit-up	r	−0.369	−0.198	−0.299	−0.351
	*p*	0.238	0.537	0.345	0.263

n = 12; * indicates significant at p < 0.05.

## 5 Discussion

The purpose of this study was to profile isokinetic knee strength in competitive Wushu Changquan athletes and to examine how these characteristics relate to agility. The results revealed velocity-dependent decreases in peak torque, dominance of extensors over flexors, and symmetrical development between limbs. Flexor–extensor ratios increased with speed, total work was greater at 240°/s for both muscle groups, and endurance ratios showed no side differences. Agility was most strongly associated with knee flexor strength and total work at 60°/s, while relative extensor torque correlated with push-up performance. The following discussion interprets these findings in relation to Changquan technical demands, biomechanical principles, and previous research.

### 5.1 Analysis of knee joint muscle strength results in competitive Wushu Changquan athletes

Isokinetic testing quantifies maximal torque across the range of motion at fixed angular velocities while the device provides adaptive resistance that matches muscular output (Tihanyi et al., 1982). The knee joint, a crucial connection in the human lower limb, has a hinge-like structure composed of the femur, patella, and tibia. Its movement primarily relies on the coordinated contraction of surrounding muscle groups, making it the primary force-bearing site during action execution. Consistent with muscle force velocity principles, both peak torque and relative peak torque were higher at 60°/s than at 240°/s for flexors and extensors, confirming that torque output decreases as contraction speed increases (Yao and Niu, 2024). This pattern is functionally relevant in Changquan that requires high-intensity knee joint involvement, particularly demanding greater extensor force during high-speed actions, jumps, and landings require the knee extensors to generate substantial force in short time frames.

The angle at which peak torque is reached reflects the optimal angle for joint force generation. Prior research indicates that the peak torque angle for knee extensors typically falls between 50 and 70° and flexor peaks near 40–60° (Pietraszewski et al., 2021). The present profiles for extensors largely fall within these ranges, whereas comparatively lower flexor outputs suggest a need to prioritize hamstring development to enhance joint stability and mitigate injury risk.

The flexor–extensor peak torque ratio is a practical indicator of agonist–antagonist balance, with commonly cited optimal ranges between 50% and 80%, and reference values of approximately 60% at 60°/s and about 83% at 240°/s (Zhiping et al., 2011; Contreras-Díaz et al., 2021). In this study, the peak torque ratio of flexor and extensor muscles in Changquan athletes remained within the reasonable range at both speeds, indicating relatively balanced strength development. Nonetheless, rapid-speed testing revealed room for improvement relative to benchmarks reported in high-level international samples. This discrepancy may stem from the frequent running jumps in Changquan routines, which tend to adaptively strengthen the quadriceps through concentric-eccentric contractions. Therefore, targeted training for posterior muscle groups like the hamstrings during rapid movements is recommended to promote synergistic development of flexor and extensor muscles and enhance stability.

Total work represents a crucial metric for assessing actual muscular work capacity (Naimo et al., 2021). Test results showed significantly higher total work output in the right knee extensors than in the left knee extensors or flexor group. This likely reflects technical lateralization in Changquan, where movements such as the “single-step kick” and several take-off actions commonly begin with the right limb, leading to greater load on the right muscle groups and consequently greater strength development (Guo and Choosakul, 2024; Wilkosz et al., 2021). Additionally, many Changquan jumping movements use the right foot as the takeoff leg, requiring powerful extensor contractions to execute the jump (Balci et al., 2021). The repetitive nature of this training leads to cumulative strength gains.

Endurance ratios reflect fatigue resistance (Morris et al., 2008). Results indicate that the right knee extensor endurance outperforms both the left knee extensors and the right knee flexors, while the left knee flexors demonstrate higher endurance compared to its extensors. This pattern is closely related to the technical structure of Changquan. For instance, in the “feint step” movement, the right foot often takes the lead as the front feint step, with the knee joint in a semi-flexed position, requiring the extensors on the right side to contract forcefully to rise (Wen and Lu, 2000). In contrast, jumping movements such as the whirling kick or aerial lotus often demand initial propulsion from the left foot, necessitating the flexors to perform a yielding action first (Chen and Tongdecharoen, 2024). This specialized training cultivates differentiated endurance adaptations.

Overall, the knee joint plays a central role in force generation and stabilizes movements during such as footwork, leg techniques, and jumps in Changquan (long fist) exercises (Liu et al., 2023). Across all indices, testing revealed no significant differences in peak torque, relative peak torque, flexor-extensor peak torque ratio, total work, and endurance ratio between the left and right knee joints, indicating balanced muscle strength development, which aligns with previous research findings (Herbawi et al., 2022). This outcome is closely related to the bilateral alternation in technical movements, such as the whirlwind kick starting with the right foot, the whirlwind turn starting with the left foot, and the mid-air lotus kick starting with both feet, all of which contribute to balanced bilateral muscle strength development. Except for slightly stronger endurance in the left flexors, all other indicators showed the characteristic of stronger extensors than flexors on the same side.

It is recommended to enhance specialized training for the flexors in practice to further improve the coordination and stability of the knee flexor-extensor groups, enhance the control and execution of technical movements, and reduce the incidence of sports injuries. The importance of interpreting strength indicators within population- and sport-specific reference frameworks has also been emphasized in recent cross-validation and normative studies, which highlight the value of relative and standardized strength measures when evaluating performance-related outcomes (Hadier et al., 2024b; Hadier et al., 2024c).

### 5.2 Analysis of agility performance in competitive Wushu Changquan athletes

Agility represents a crucial aspect of an athlete’s overall physical fitness and directly impacts the quality of technical execution in complex movement sequences (Lei and Lv, 2022). Enhanced agility improves coordination and fluidity during movement transitions, playing a particularly vital role in competitive wushu routines. According to training manuals, male athletes who complete the T-test in less than 9.5 s demonstrate excellent agility standards (Negra et al., 2017). Current study test results indicate that Changquan athletes demonstrated overall strong performance in the T-test, confirming their high agility levels, indicating strong change-of-direction capacity.

Changquan demands rapid transitions in direction and posture within high-velocity sequences (Dai, 2016). Prior analyses identify lower-limb strength, movement technique, and body composition as key determinants of agility development (Brughelli et al., 2008). Modern competition intensifies the requirement for rapid movement reaction, precise body control, and efficient movement orientation in addition to technical skill. The technical structure of Changquan is complex, involving movements such as stepping into a side somersault, striking into a flying kick, a single back kick followed by a smashing punch, or a spinning kick transitioning into a horse stance. These sequences often encompass multiple directional shifts and posture transitions. Such movements are not mere repetitions but comprehensive tests of an athlete’s ability to link actions seamlessly and react with rapid physical response. Particularly in jumping techniques, the instantaneous takeoff after a run-up demands swift energy conversion, placing high demands on bodily control.

Therefore, agility is not only the foundation for executing technical movements but also a crucial factor in showcasing the aesthetic appeal and style of the performance in Wushu. Changquan athletes should scientifically plan the development of agility throughout their training, integrating it into all phases of their regimen (Cao et al., 2025). Through specialized drills, they can continuously enhance their ability to change direction, react to movements, and control rhythm, thereby refining their technical execution and elevating their competitive level.

### 5.3 Associations between knee joint strength and agility qualities

Muscle strength forms the foundation for athletes to execute technical movements (Ríos, 2025). As a crucial joint in the lower limbs, the knee’s flexion and extension play an especially vital role in Changquan training and competition (Liu et al., 2023). Multiple muscle groups collectively stabilize the knee joint and facilitate movement, with the quadriceps serving as the primary extensors and the hamstrings, sartorius, and gastrocnemius acting as flexors (Guo and Choosakul, 2024). In Changquan disciplines, movements like lunges, horse stances, and high-difficulty jumps including whirlwind kicks, spinning twists, and aerial outer-swinging lotus kicks all require knee joint power to handle multiple tasks such as takeoff, shock absorption, and directional changes (Wen and Lu, 2000; Cao et al., 2025).

The present findings highlight a significant positive association between knee flexor peak torque and T-test performance, indicating flexor strength significantly enhances agility. This observation aligns with previous research suggesting that knee flexor capacity contributes substantially to rapid deceleration and direction change during dynamic movements (Brughelli et al., 2008). Moreover, total work of the right knee flexors at 60°/s exhibited a strong positive correlation with T-test performance, suggesting that high work capacity at slower angular velocities underpins effective braking and reacceleration phases. In technical actions such as running-up roundhouse kicks or spinning back kicks, athletes must perform eccentric braking followed by rapid concentric acceleration, mirroring the neuromuscular demands of the T-test. Thus, superior flexor work capacity appears integral to maintaining control and speed during directional transitions in Changquan (Cao et al., 2025).

During Changquan practice, frequent transitions between movements such as rapidly transitioning from a standing position to a squat, followed by a bow stance or horse stance rely on the strength of the knee flexors to maintain body control and execute movements smoothly (Yessis, 2006). Long-term training emphasizing these transitions likely induces adaptive increases in hamstring strength and coordination, promoting smoother motion linkage and quicker responses during complex sequences.

The relative peak torque of the right knee extensors at 60°/s and 240°/s showed a strong correlation with push-up test performance, indicating that extensor strength is crucial for executing movements like squat-to-jump transitions (Chen et al., 2023). For instance, rising from a horse stance into a jump or rotational movement requires powerful extensors to facilitate the transition, establishing optimal muscle initial length and tension for the subsequent action (Guo and Choosakul, 2024). With accumulated training, the knee extensors progressively strengthen, gradually establishing a force generation pattern adapted to the Changquan style.

The flexor–extensor peak torque ratio on the right side at 60°/s demonstrated a significant negative correlation with push-up scores, reflecting mild extensor dominance among athletes. Excessive dominance of the extensors may compromise joint stability and elevate injury risk. Therefore, balanced development between flexors and extensors should remain a central objective of strength conditioning programs.

Although the correlation between peak torque angle and agility was moderate and non-significant, the trend suggests that athletes capable of producing peak torque at more functional joint angles may display more efficient movement control. Similarly, the moderate positive correlation between left knee extensor endurance ratio and T-test performance implies that endurance capacity supports sustained agility performance during longer or repetitive routines. Given that Changquan features frequent combinations of high-intensity strikes, jumps, and turns, both muscular strength and endurance contribute to maintaining stability and execution quality (Li et al., 2025).

In summary, the results affirm that knee muscle strength particularly hamstring torque and work capacity plays a critical role in enhancing agility. Agility, in turn, represents the functional manifestation of neuromuscular coordination and rapid strength utilization. Training programs for Changquan athletes should therefore emphasize balanced development between flexors and extensors, with a focus on eccentric hamstring strength, dynamic stability, and muscular endurance to support fluid movement transitions, injury prevention, and superior technical expression. Coaches are advised to prioritize balanced development of both flexor and extensor muscle strength in training programs. This approach enhances muscle group coordination, avoids overemphasizing any single quality, and promotes comprehensive physical development. Such a foundation will lay solid groundwork for elevating the competitive performance of Changquan athletes.

### 5.4 Limitations

This study is not without limitations. Although all eligible athletes within the training cohort were invited to participate, thereby minimizing selection bias related to performance level, the cross-sectional design limits causal inference. In addition, the small sample size and exclusive focus on male elite Wushu Changquan athletes may restrict the generalizability of the findings to other populations. Future longitudinal studies involving larger and more diverse samples, as well as objective monitoring approaches, are warranted to strengthen causal interpretation and external validity.

### 5.5 Research recommendations

While conducting knee joint strength training, it is essential to regularly monitor the bilateral knee joint muscle strength and the balanced development of knee flexor and extensor muscles. If discrepancies are observed, targeted strength training for the weaker side can be implemented to enhance the athlete’s physical performance and prevent sports injuries. The experimental subjects of this study were elite male Changquan athletes. Due to the distinct movement characteristics of Changquan compared to Nanquan and Taijiquan, as well as the significant differences in muscle strength levels between male and female athletes, it is recommended that future research focus on female athletes or athletes from different disciplines to develop more targeted training programs. The completion of Changquan technical movements relies on the coordinated action of various joints. This study only preliminarily explores the relationship between knee joint muscle strength and agility, and future research could focus on the relationship between muscle strength and agility in other joint areas.

## 6 Conclusion

Competitive Wushu Changquan athletes demonstrated relatively equal bilateral knee strength, although flexor strength was consistently lower than extensor strength. Isokinetic knee muscle strength was associated with agility performance, with flexor maximal and explosive strength showing the strongest relationships with change-of-direction ability, while extensor maximal strength was more closely related to movement transitions and technical execution. Among the three strength components analyzed, the degree of correlation with agility followed the order: maximal strength > explosive power > strength endurance. These findings highlight the functional importance of lower-limb strength characteristics in supporting agility-related performance demands in elite Changquan athletes.

From a sport-specific perspective, training programs may benefit from emphasizing balanced development of knee flexor and extensor strength, particularly targeted enhancement of flexor capacity, to support movement control and technical efficiency in high-level Changquan performance. The implications of these findings are limited to elite athletic training contexts, and further longitudinal research is required to examine causal relationships and broader applicability.

## Data Availability

The raw data supporting the conclusions of this article will be made available by the authors, without undue reservation.
