# Supplementary material for: A study on the correlation between knee muscle strength and agility in competitive Wushu Changquan athletes
Source: Front Physiol. 2026 Feb 11;17:1736668. doi: 10.3389/fphys.2026.1736668 (PMC12932155; doi:10.3389/fphys.2026.1736668)
Supplement: Supplementary file 1 [file Supplementaryfile1.docx]

**Appendix A**

## **Section 1: Expert Interview Outline**

Dear Experts,

Hello! I am a master's student at Beijing Sport University, currently conducting research for my thesis. Thank you for taking the time to participate in this interview. The information collected will be used solely for academic research purposes. All personal data will remain strictly confidential. Your valuable insights and support are sincerely appreciated.

This interview aims to gather professional opinions from both Physical Fitness Specialists and Wushu Taolu Experts to enhance the scientific rigor and practical relevance of the study on the correlation between knee muscle strength and sensitivity in competitive Wushu (Changquan) athletes.

**Section A: Questions for Physical Fitness Specialists**

1. What indicators do you consider most appropriate for assessing knee muscle strength?
2. In your opinion, what is the importance of knee muscle strength for martial arts athletes?
3. Which test methods do you believe are most suitable for measuring sensitivity (agility and reaction qualities)?
4. Do you believe there is a correlation between knee muscle strength and sensitivity? If so, how would you describe this relationship?
5. What key considerations should be considered during the testing process to ensure accuracy and safety?

**Section B: Questions for Wushu Taolu Experts**

1. To what extent do you think knee muscle strength can influence the performance level of Changquan (Long Boxing) athletes?
2. What role does sensitivity play in the technical execution and performance of Changquan athletes?
3. What are the distinctive characteristics of sensitivity in Changquan performance, and what testing methods would you recommend assessing it effectively?
4. What practical or procedural issues should be carefully considered during the testing process for Wushu athletes?

## **Section 2: Questionnaire (Test Indicators Selection)**

Dear experts,

Hello! I am a master's student at Beijing Sport University, and I am currently writing a thesis. In order to improve the scientific validity and rationality of the research, I would like to identify the most appropriate knee muscle strength and (agility) testing methods. I sincerely hope you will provide an objective evaluation through this questionnaire. Your responses will be used solely for research purposes. Thank you very much for your time and support.

Please tick “√” in the appropriate option. In addition to the indicators listed below, if you believe there are other suitable sensitivity test indicators, please specify them in the blank space provided.

**Name:**

**Job title:**

**Workplace:**

**Part 1: Knee Muscle Strength Test Indicator Screening**

| **No** | **Test Metric** | **Agree to Add** | **Agree Not to Add** |
| --- | --- | --- | --- |
| 1 | Isokinetic Test (e.g., 60°/s, 180°/s) | ☐ | ☐ |
| 2 | Isometric Knee Extension Test | ☐ | ☐ |
| 3 | 1 RM Leg Press | ☐ | ☐ |
| 4 | Handheld Dynamometer Test | ☐ | ☐ |
| 5 | Vertical Jump Test (as indirect power indicator) | ☐ | ☐ |

**Additional indicators you recommend:**

**Part 2: Sensitivity (Agility) Test Indicator Screening**

| **No** | **Test Metric** | **Agree to Add** | **Agree Not to Add** |
| --- | --- | --- | --- |
| 1 | 505 Test | ☐ | ☐ |
| 2 | T-Test | ☐ | ☐ |
| 3 | Illinois Agility Test | ☐ | ☐ |
| 4 | Burpees Test | ☐ | ☐ |
| 5 | Hexagon Jump | ☐ | ☐ |
| 6 | Turn-Back-and-Run | ☐ | ☐ |

**Additional indicators you recommend:**

## **Section 3: Basic Information of the Participants**

| No | Height (cm) | Weight (kg) | Age (years) | Years of training (years) |
| --- | --- | --- | --- | --- |
| Subject 1 | 170 | 63 | 23 | 18 |
| Subject 2 | 174 | 65 | 21 | 12 |
| Subject 3 | 179 | 66 | 22 | 14 |
| Subject 4 | 170 | 63 | 23 | 14 |
| Subject 5 | 179 | 78 | 23 | 12 |
| Subject 6 | 170 | 65 | 21 | 11 |
| Subject 7 | 170 | 62 | 18 | 12 |
| Subject 8 | 170 | 70 | 27 | 20 |
| Subject 9 | 165 | 57 | 20 | 12 |
| Subject 10 | 170 | 60 | 23 | 15 |
| Subject 11 | 177 | 65 | 21 | 14 |
| Subject 12 | 174 | 68 | 19 | 11 |
